# Silk fibroin micro-particle scaffolds with superior compression modulus and slow bioresorption for effective bone regeneration

**DOI:** 10.1038/s41598-018-25643-x

**Published:** 2018-05-08

**Authors:** Anuya Nisal, Raeesa Sayyad, Prachi Dhavale, Bhakti Khude, Rucha Deshpande, Vidhyashri Mapare, Swati Shukla, Premnath Venugopalan

**Affiliations:** 1 Polymer Science and Engineering Department, National Chemical Laboratory, Pune, 411008 India; 2 BiolMed Innovations Pvt. Ltd., 100, NCL Innovation Park, Dr. Homi Bhabha Road, Pune, 411008 India

## Abstract

Silk fibroin (SF), a natural polymer produced by *Bombyx mori* silkworms, has been extensively explored to prepare porous scaffolds for tissue engineering applications. Here, we demonstrate, a scaffold made of SF, which exhibits compression modulus comparable to natural cancellous bone while retaining the appropriate porosities and interconnected pore architecture. The scaffolds also exhibit high resistance to *in-vitro* proteolytic degradation due to the dominant beta sheet conformation of the SF protein. Additionally, the scaffolds are prepared using a simple method of microparticle aggregation. We also demonstrate, for the first time, a method to prepare SF micro-particles using a Hexafluoroisopropanol-Methanol solvent-coagulant combination. SF microparticles obtained using this method are monodisperse, spherical, non-porous and extremely crystalline. These micro-particles have been further aggregated together to form a 3D scaffold. The aggregation is achieved by random packing of these microparticles and fusing them together using a dilute SF solution. Preliminary *in-vitro* cell culture and *in-vivo* implantation studies demonstrate that the scaffolds are biocompatible and they exhibit the appropriate early markers, making them promising candidates for bone regeneration.

## Introduction

Bone regeneration remains to be an active and flourishing area for research today^1–4^. Appropriate substrates or scaffolds form an integral part of tissue engineering and regenerative therapies. For a substrate to effectively perform as a scaffold, the following characteristics are vital. Most importantly, the scaffold must be biocompatible, which means that the scaffold must support the cellular activity and the scaffolding material and its biodegradation products, if any, must elicit only minimal inflammatory response^5,6^. The scaffold must be porous and must support production of natural extracellular matrix and allow efficient transport of gases and nutrients and migration of cells. If the material is biodegradable, the biodegradation rate of scaffold must be in accordance with that of the new tissue regeneration^1^. For bone regeneration, the scaffold must have excellent stiffness to provide structural strength. Polymeric scaffolds providing the appropriate physical, chemical and mechanical cues for tissue regeneration have thus, been extensively studied^6^. The choice of the scaffolding material provides the chemical cues for the cellular activity. Biodegradable natural polymers are especially a promising alternative as the need for surgical removal is obviated and they are chemically similar, usually proteinaceous, to the natural extra-cellular matrix.

One such naturally derived polymer, Silk Fibroin (SF) has been extensively explored for regenerative applications in the last few decades^7–10^. SF is a natural protein polymer extracted from the cocoon of silkworm *Bombyx mori*. It has excellent and proven biocompatibility, outstanding thermo-mechanical stability, tunable biodegradation and ease of processability^11,12^. Several studies have successfully demonstrated use of SF for vascular, neural, bone, ligament, cartilage, skin, intervertebral disc, heart, ocular and spinal cord tissue regeneration^7,8,11,13^. A variety of different cell lines including mesenchymal stem cells, fibroblasts, osteoblasts, myoblasts, chondrocytes, keratinocytes and neurons have been cultured on these SF matrices. These studies suggest that the SF matrix provides the appropriate chemical cues required for regeneration.

Also, as discussed earlier, the physical and mechanical properties of the scaffold also affect the performance and these factors are primarily governed by the design of the scaffold. The ease of processability has resulted into a variety of forms of SF scaffolds such as hydrogels, sponges, and non-woven mats. Many techniques such as salt leaching, gas foaming, extrusion layering, additive manufacturing or freeze-drying and their modifications and/or combinations have been reported to fabricate 3D SF porous architectures^14^. Recently, SF scaffolds with higher load bearing capacity have been developed^15,16^. However, these scaffolds have random pore sizes and limited mechanical properties (Dry compression modulus ~50 MPa). The wet compression modulus values for these scaffolds are either poor or have not been reported. Thus, there is a need to further develop SF scaffolds with improved mechanical properties.

Of the many types of 3D porous polymeric scaffold designs, microparticle or microsphere scaffolds have found great utility, especially in bone regeneration^17^. Polymeric microparticles can be produced by a variety of techniques including emulsification, spray drying, coacervation, grinding, electro-spraying, etc.^17^. The particles are then aggregated in a random packing conformation and fused together to form the 3D scaffold. Microparticle fusion is achieved using heat sintering, solvent/non-solvent sintering, solvent vapor technique,etc.^1^. The scaffolds so obtained are characterized by interconnected pores, controlled pore size, limited porosity and excellent mechanical properties. This bottom up approach also allows better spatial distributions of functionalities in the scaffold^18^. The pore size can be controlled by changing the microparticle size. The porosity and mechanical properties can be tuned by changing the microparticle fusing parameters such as sintering time and temperature. The bulk porosities typically observed in these scaffolds range from 30–50%, as is expected from monodisperse randomly packed spheres^19^. A variety of natural^20^ and synthetic^18,21^ polymers, their blends^1^, composites with various fillers^19^, and functional bioactive compounds have been used to prepare microparticle scaffolds and the properties of the scaffolds so obtained are found to be interesting especially for bone tissue regeneration.

Silk fibroin microspheres or microparticles have been prepared using various techniques such as water-in-oil emulsion, electrostatic fields, lipid templating, spray drying, salting out, break up of laminar aqueous jet, etc. and this literature has been extensively reviewed^22–25^. Except for the laminar flow break-up method, the particles produced using all other techniques are in the range of 0.2–20 µm and are thus found to be particularly useful in drug delivery applications. Only authors Qu *et al*.^24^ have studied SF microspheres for applications in tissue engineering. It is also interesting to note that, inspite of the several advantages of 3D microparticle scaffolds; there is no report to prepare 3D microparticle SF scaffolds for use in bone regeneration.

Here, we report a novel and patented method for preparation of SF microparticles by coagulating droplets of SF-Hexafluoroisopropanol (HFIP) in a methanol bath^26^. Although the HFIP-methanol solvent-coagulant combination has been used for artificial fiber spinning for SF^27^, this method has not been reported for the production of monodisperse, spherical and crystalline SF microparticles. The microparticles so obtained were then aggregated together in random packing and dilute SF solution was used as a glue to fuse these particles together as per the protocol described in Nisal *et al*.^28^. The 3D scaffold so obtained is porous, biocompatible, has interconnected porosity and an excellent resistance to proteolytic degradation. Most importantly it has mechanical properties comparable to natural cancellous bone.

## Results and Discussion

A 3wt/v% SF + HFIP solution was used to prepare SF micro-particles. It is observed that the micro-particles are formed continuously using this protocol. The micro-particles obtained are spherical in shape. Image analysis results, done on the optical micrographs, show that the microparticles have an average size of 503 μm with a standard deviation of 0.02%. The d_10_, d_50_ and d_90_ values obtained from the image analysis results are 480, 506, and 543 respectively. These values result in a d_90_/d_10_ ratio of 1.13, indicating monodisperse SF particles. The sphericity value as obtained from the image analysis using ImageJ software was found to be 0.96. Here, a value close to 1 is attributed to a completely spherical particle. These results imply that the method can be used to produce extremely mono-disperse spherical particles.

**Figure 1:**
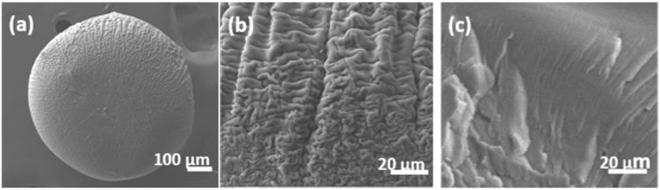
Represents SEM images of (**a**) particle (**b**) particle surface and (**c**) Cryo-fractured core of the particle.

The particles were further characterized using scanning electron microscopy to observe the surface and the core morphology. As can be seen in the Fig. 1(a), the electron micrograph confirms the sphericity of the particle observed in the optical micrograph. However, the higher magnification images of the surface of the microparticle exhibit a very peculiar morphology (Fig. 1(b)). Although the HFIP-Methanol solvent-coagulant system has been used earlier for production of other forms of SF such as film, fibers and 3D scaffolds^27^, this morphology consisting of folds and ridges has not been reported. Also, it has been reported earlier, that higher surface roughness promotes better adhesion of cells. However, for SF scaffolds surface roughness has not been found to be dominant factor influencing adhesion^29,30^. Thus, *in-vitro* cell culture studies on these scaffolds will be necessary to elucidate the effect of this factor on cell adhesion and proliferation. Additionally, the micrographs provide a clear evidence for absence of surface micro-porosity. Further, high-resolution microscopy studies may be necessary to probe the nano-features/pores present on the surface of these particles. Similarly, the core of the microparticle exhibits the absence of micro-porosity, as is evidenced from the cut section image shown in Fig. 1(c). Thus, it may be concluded here that the micro-particles so produced have negligible intra-particle porosity.

**Figure 2:**
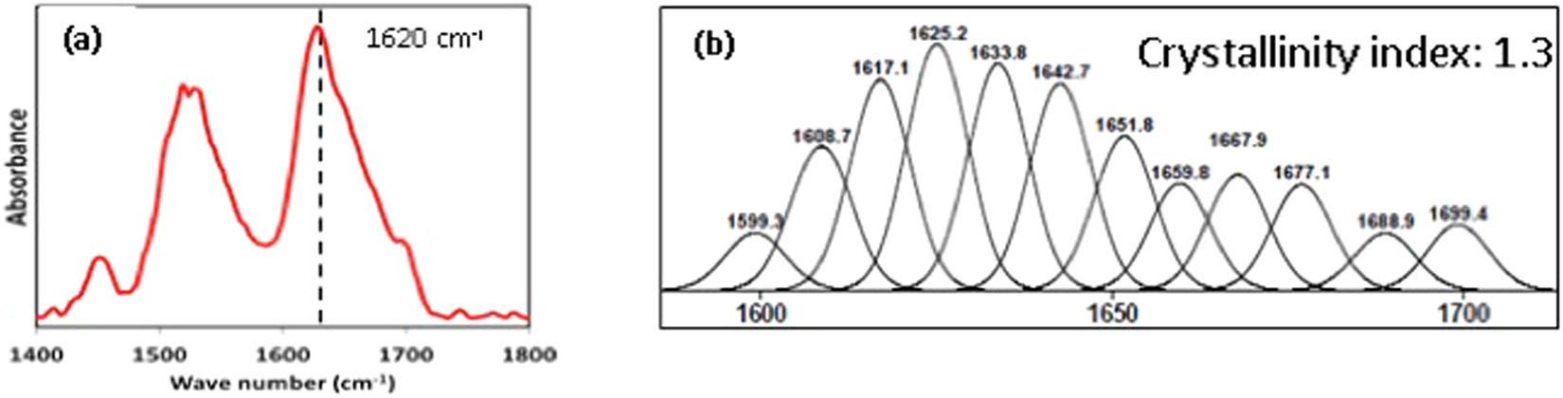
(**a**) ATR Spectra between 1400–1800 (Amide I region). (**b**) Deconvoluted Amide I region.

FTIR spectroscopy has been routinely used to quantify the secondary protein structure of SF^31^. We did an ATR spectroscopic study on the SF micro-particles to understand the SF protein conformation. As can be seen in the Fig. 2(a), the Amide I region shows a peak around 1620 cm^−1^. This is characteristic of a dominant beta sheet conformation in the protein. Similar results of obtaining a predominant beta sheet structure when HFIP is used as a solvent have also been reported by Zhao *et al*.^27^ Deconvolution of the Amide I peak, in Fig. 2(b), as per the peak positions identified by Hu *et al*.^31^, estimates that the particles have a crystallinity index of about 1.3. Here, the crystallinity index greater than 1 indicates predominance of beta sheet structures in the protein.

Thermogravimetric analysis was done on the micro-particles. The graph shows characteristic two-step decay, with first onset degradation temperature of 214 °C and peak degradation temperature of 284 °C. These values are in agreement with those reported in the literature for highly crystalline SF scaffolds^32^. This study further support the FTIR results discussed earlier and corroborate the crystalline nature of SF in the micro-particles so obtained. (See Supplementary Fig. S1).

Thus, the data obtained from optical microscopy, electron microscopy, infrared spectroscopy and thermogravimetry suggests that the use of a solvent-coagulant combination of HFIP and methanol for SF results in the formation of mono-disperse, spherical, non-porous and highly crystalline SF micro-particles.

**Figure 3:**
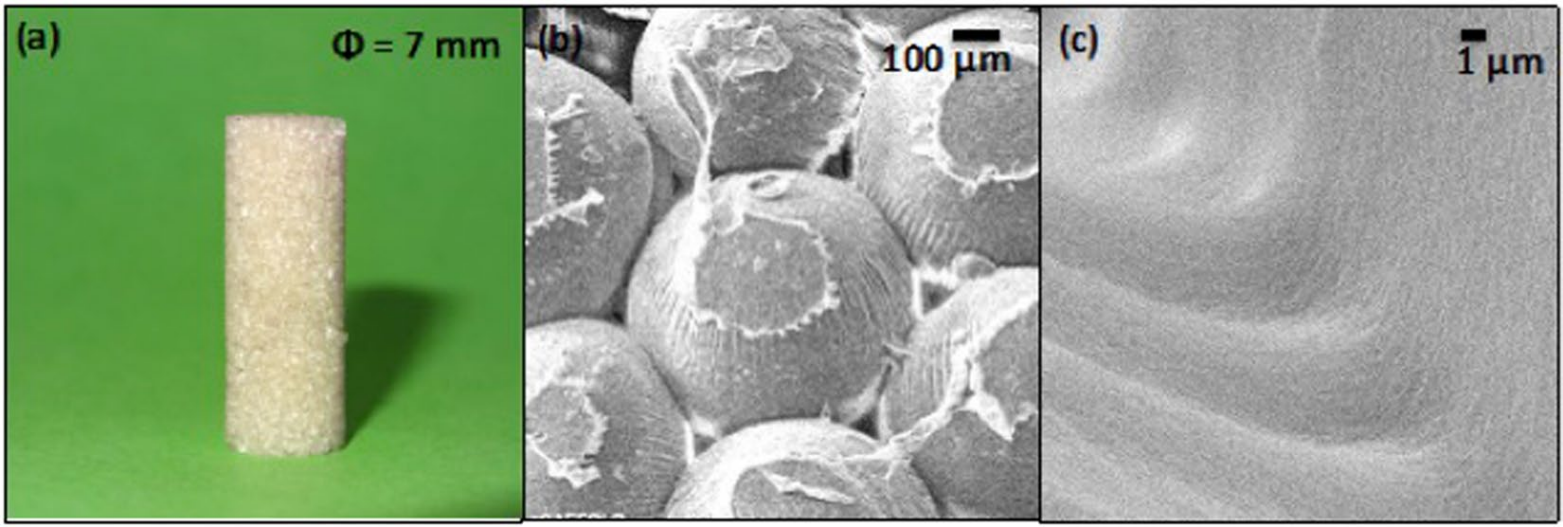
(**a**) Representative 7 mm diameter cylindrical scaffold prepared using SF micro-particles particles; (**b**) SEM micrograph of scaffold showing mono-disperse SF micro-particles arranged in a hexagonal packing structure. (**c**) Magnified image showing line of fusion between two particles.

The SF microparticles were then fused together using dilute RSF as per the novel and patented protocol developed in our lab^28^. A cylindrical mold was used for demonstration purposes. However, it may be noted here that since the process involves filling up a mold with the microparticles the procedure can be used to prepare more complex and customized shapes. As can be seen in Fig. 3(a), a cylindrical scaffold was formed by using the process. The scaffold has several interesting and useful features. The higher magnification SEM picture, Fig. 3(b), reveals that the monodisperse spherical SF particles exhibit a hexagonal packing structure. This architecture was observed in most places throughout the scaffold. However, few defects were also observed in this packing. (See Supplementary Fig. S2). This is anticipated since the microparticles were filled into the mold and the mold was tapped to improve packing of these spheres. This results in randomly packed architecture. Figure 3(c) shows the interface between two microparticles fused together using the SF solution as glue. Different methods have been reported in the literature for fusion of polymeric microparticles to form 3D scaffolds. The most commonly used methods involve heat sintering and solvent fusion^18^. SF is a protein polymer and does not exhibit a melting transition. Thus, heat sintering is clearly not an alternative. Also, SF has exceptional chemical resistance; this implies that the protein polymer has poor or negligible solubility in most organic solvents^33^, especially in its highly crystalline form. Use of dilute regenerated SF solutions as a glue to fuse other SF architectures or forms has not been reported. Thus, we have demonstrated a novel method to produce SF microparticle scaffolds. The scaffolds so obtained have been characterized for porosity, compression modulus - both in wet and dry conditions, accelerated degradation in proteolytic environment and also for their biological properties using *in vitro* and *in vivo* techniques.

**Figure 4:**
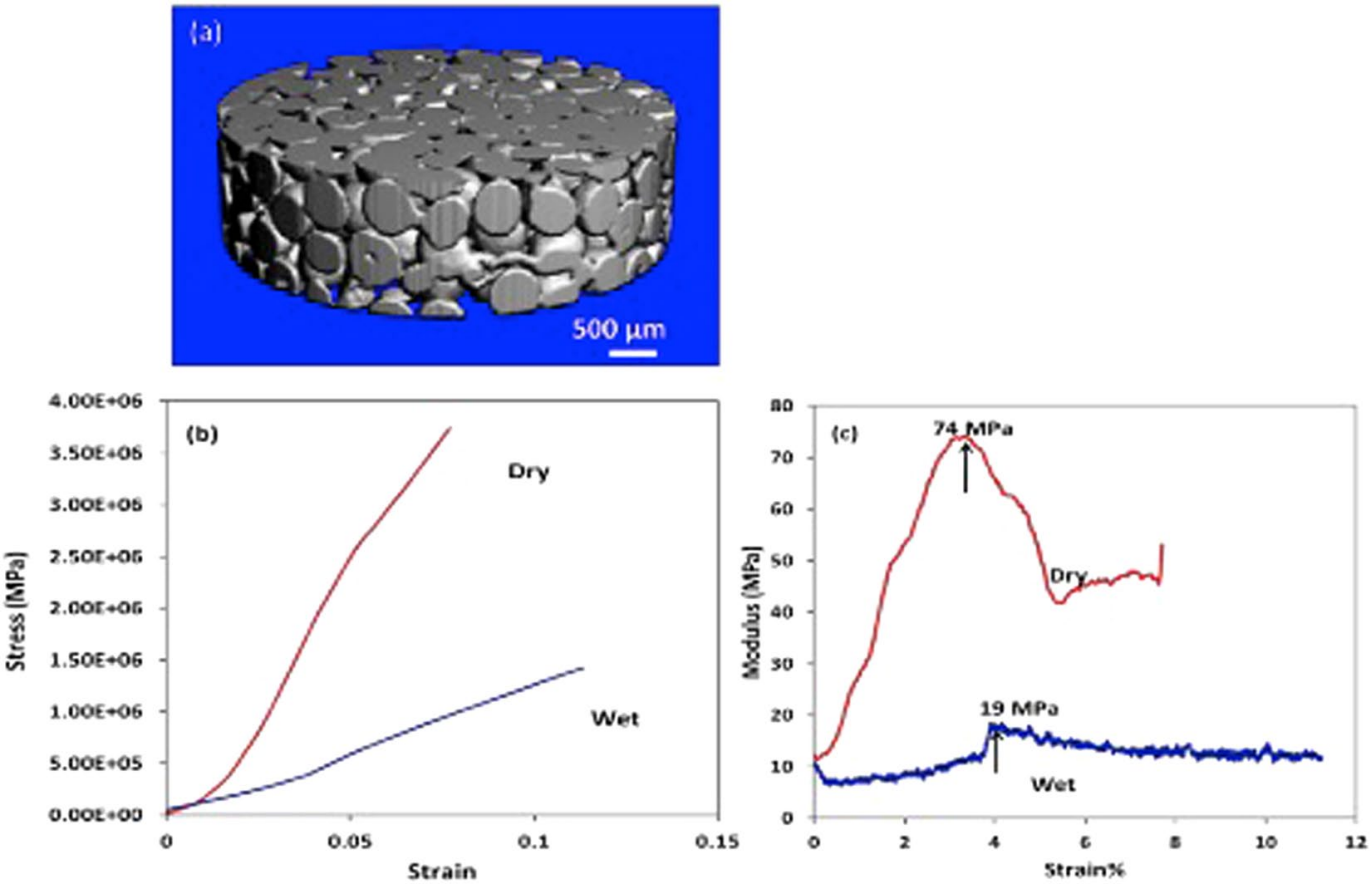
(**a**) X-ray tomography image of cylindrical section of scaffold showing interconnected pores, pore size and estimating bulk porosity. (**a**) Typical stress-strain curve for dry and wet compression mode measurement of SF scaffold and (**b**) Modulus vs % Strain plot.

The porosity of the scaffolds was characterized using two methods. A simple lab protocol was used to estimate the bulk porosity of the scaffold by measuring the dimensions of the scaffold and calculating the volume, accurately weighing the scaffold and using the reported density of SF^34^. It was found that the scaffolds exhibited an average porosity of 42 ± 2%. These laboratory results were further validated using micro X-ray computed tomography imaging (micro CT). Micro CT has emerged as a promising method for visualization and quantification of the 3D architecture of a scaffold^35^. It has been used to provide information about porosity, pore size and pore wall thickness. Micro CT results on our scaffolds (Fig. 4(a)) suggested a bulk porosity of 38.5%. This corroborates the porosity measured by the theoretical method presented above. Also, it is worthwhile to note here that random packing of monodisperse spheres typically results in porosities of the order of 46%; however the randomness of packing is still an ill-defined term. Also, as the randomness reduces, there is an increase in the packing order in the system. This increase in packing order causes further reduction in porosity and a maximum achievable porosity is about 25%^36^. Thus, it may be concluded here that the SF microparticles in the scaffold exhibit slightly higher order in packing as compared to the maximally random jammed state.

The pore sizes were found to be in the range of 0–275 μm, with an average pore size of 138 μm. The pore size distribution is found to be fairly narrow (See supplementary Fig. S3). The pore size values obtained from Micro CT are comparable to those observed in SEM micrographs. The interconnectivity of the pores can also be seen and confirmed from the micro CT image reported here in Fig. 4(a). It is important to note here that this large pore size is a manifestation of the large particle diameters used in preparation of the scaffolds unlike most other reports discussed in the introduction section where particle sizes are primarily in the range <20 μm. The large pore size and interconnected porosity obtained using this microparticle scaffold will enable free migration of cells and transport of nutrients.

The mechanical properties of the scaffolds are typically useful to further identify their applicability in specific tissue engineering domains. For example, scaffolds to be used for bone tissue engineering must exhibit superior mechanical properties as compared to those being used for soft tissue reconstruction. We report here the compression modulus of these SF microparticle scaffolds both in the dry and wet state. As can be seen in Fig. 4(b), the stress strain graph for both the wet and dry scaffolds displays a change in slope throughout the measurement. This means that the modulus of the scaffold is continuously changing as a function of strain. A similar observation has also been reported for uniaxial compression measurements conducted on natural cancellous bone. Thus, an alternative method has been proposed to report the modulus of the scaffolds. Here, we report the modulus as the peak in the modulus vs strain graph as proposed by Li *et al*.^37^. The compression modulus for the SF microparticle scaffold was found to be 70 ± 6 MPa in the dry state and 18 ± 2 in the wet state, as shown in Fig. 4(c). The compression modulus reported here is significantly higher than other unfilled SF scaffolds that have been reported in the literature^15,16^. This property makes these scaffolds highly suitable for bone tissue engineering applications.

**Figure 5:**
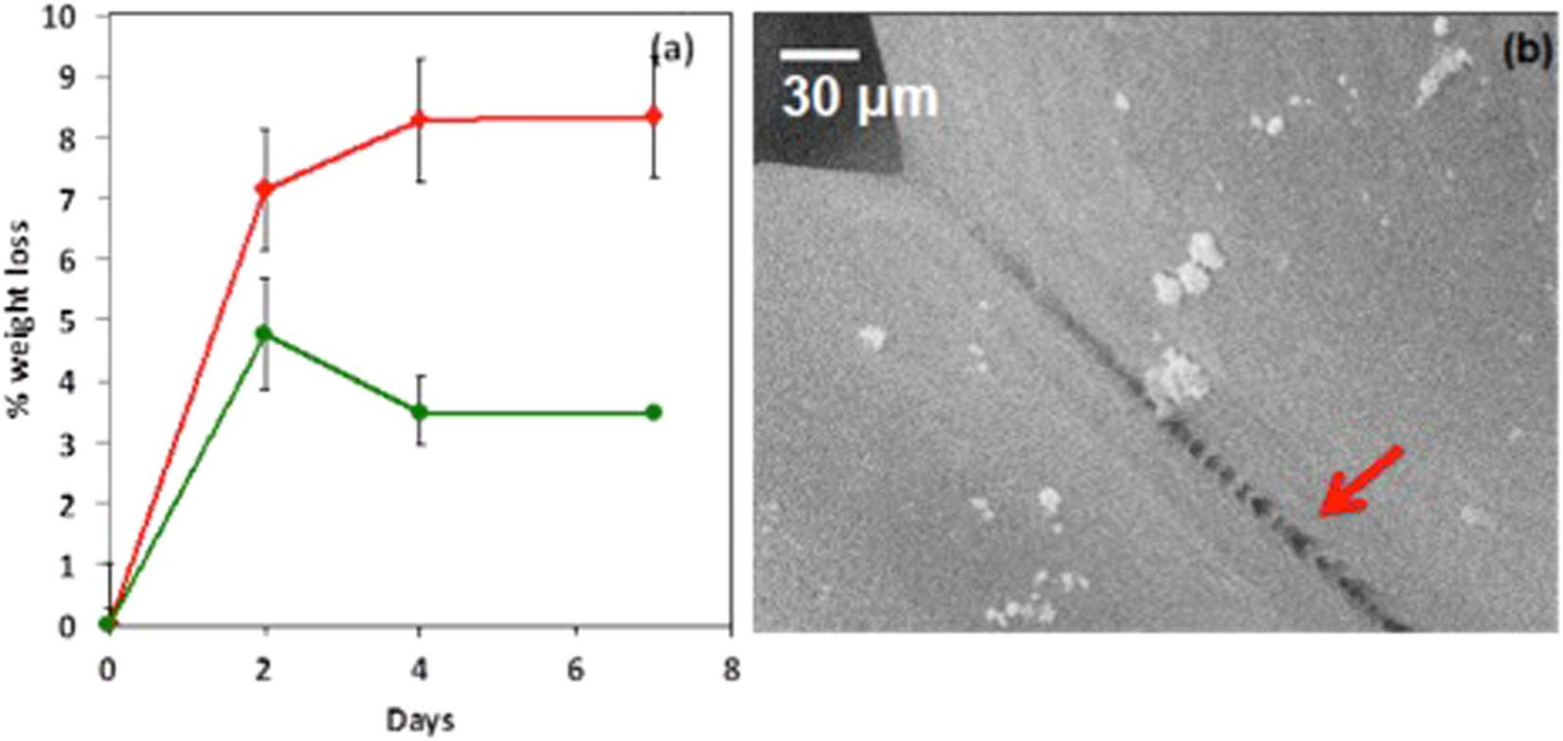
(**a**) Weight loss measurements for enzymatic bioerosion (weight loss) for scaffolds with degummed SF fibers as control. (**b**) SEM image showing weakening of fusion line between two particles after enzymatic degradation for 7 days.

Bioerosion or *in-vivo* bioresorption of the scaffolds is an important property for tissue engineering applications. The rate of bioresorption of the scaffold must be similar to the rate of growth of neonatal tissue. Thus, we characterized the rate of bioresorption for our microparticle scaffold by doing a proteolytic degradation experiment *in-vitro*. Since there is significant body of literature available on *in-vivo* degradation of degummed silk fibers, we used these as a control for our experiment^38^. As can be seen in Fig. 5(a), the weight loss observed in proteolytic degradation experiment was only slightly higher than that observed for the degummed SF fibers, indicating significant resistance to degradation. This is expected since the IR data presented earlier, did show that the SF microparticles are highly crystalline in nature. It has been very well documented that increase in beta sheet content leads to longer degradation time for SF. We also probed the morphology of this 7 day degraded scaffold using scanning electron microscopy. Interestingly, we observed that the degradation of the scaffold was initiated at the location of fusing of the two particles, as shown in Fig. 5(b). The surface of the particles did not show measurable change in surface topology due to formation of pits/crazes or crevices for the duration of this experiment.

**Figure 6:**
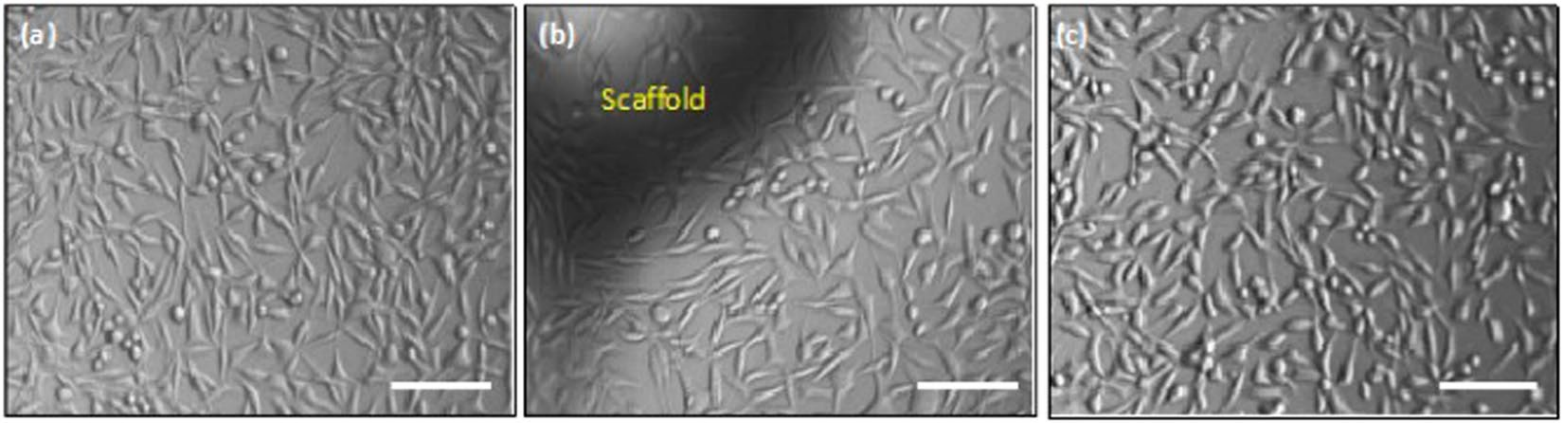
*In vitro* cell viability studies for (**a**) culture plate control (**b**) in direct contact with SF scaffold (**c**) extract contact using L929 mouse fibroblast cell line. Scale bar is 100 μm.

The biocompatibility of these SF microparticle scaffolds was evaluated as per the *in-vitro* protocols described in standard test method – ISO 10993–5. This test is a preliminary, but mandatory, screening for a material to be intended for use as an implantable device. Both the direct contact, as well as extract contact, experiments were conducted in triplicate and the results have been summarized in Supplementary data. (See supplementary Table S1) and Fig. 6(a). The % viability of the cells, measured using the tissue culture plate control, was always found to be >85%. As per the ISO guidelines, a material exhibiting more than 70% viability can be considered to be non-cytotoxic and can be further evaluated for toxicity using *in-vivo* experiments. Also, the cell morphology observed in Fig. 6(b,c) corroborates the non-cytotoxicity of SF microparticle scaffold since the cell morphology is unaltered as compared to the control. The *in-vitro* cytotoxicity studies were followed by *in-vivo* subcutaneous implantation in SD rats (See Supplementary Fig. S4), which further provide support for the biocompatibility of SF scaffold.

**Figure 7:**
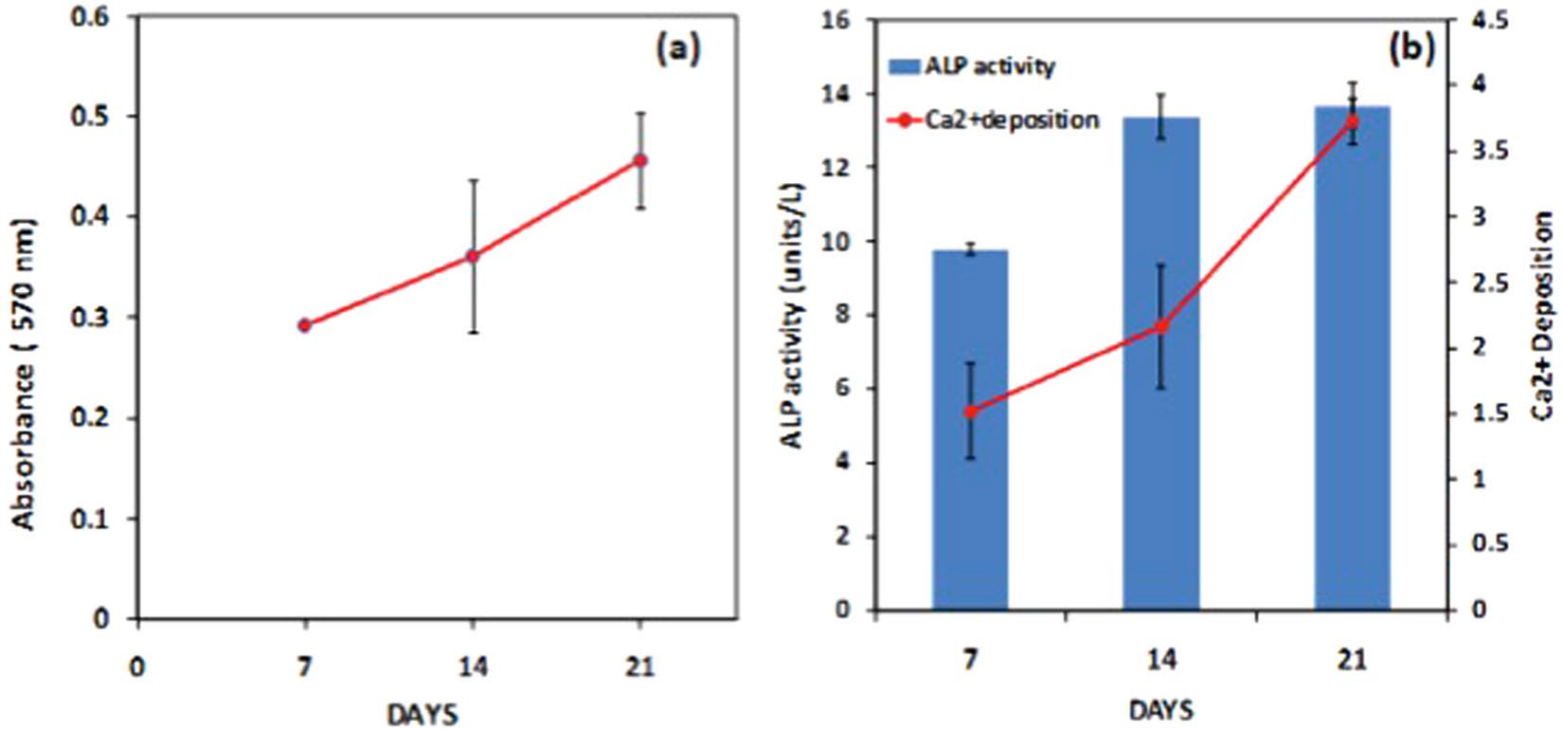
(**a**) MTT Proliferation (**b**) ALP activity and Ca^2+^ deposition (by Alizarin Red S Staining).

MG63 (an osteosarcoma cell line) was used to assess potential of SF scaffold to support osteoblast differentiation and proliferation. Cells cultured in osteoblast differentiation medium show increase in absorbance at 570 nm indicating proliferation of cells on days 7, 14 and 21 (Fig. 7(a)). These results support the fact that SF-scaffold provides suitable environment for growth and proliferation of osteoblast cells.

Osteoblasts are the major cells type participating in new bone formation. They actively participate in bone matrix synthesis and bone mineralization. Thus, we studied two important markers of physiological function of osteoblasts on SF scaffold *in-vitro*. These two parameters are ALP activity and deposition of Ca^2+^ (early to late markers of osteoblast differentiation). Time dependent increase in ALP expression and Ca^2+^ deposition was observed (Fig. 7(b)). Both these results corroborate with our proliferation and viability data, implying the suitability of SF scaffold as an environment for proliferation and differentiation of pre-osteoblast cells in mature osteoblasts. Also, we have recently published more *in-vitro* data on our SF microparticle scaffolds for specific use in bone tissue engineering^39^. Our results show that SF microparticle scaffold support cell adhesion, proliferation and mineralization of MG63 osteoblast-like cells.

**Figure 8:**
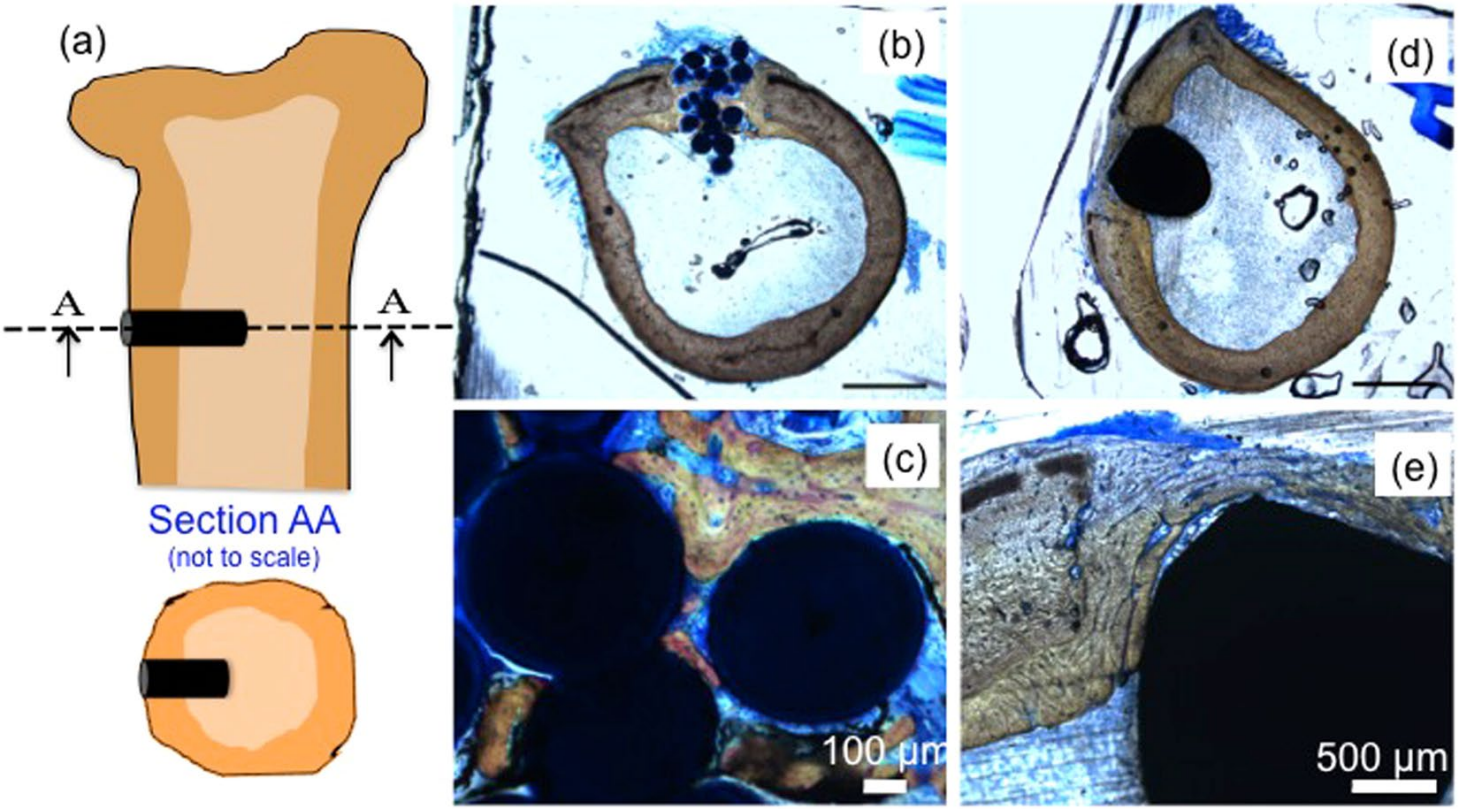
(**a**) Schematic representation for bone implantation in rabbit femur and sections for histopathlogical studies (**b**) low and (**c**) high magnification images for histopathological sections for SF microparticle scaffold and positive control using titanium pins (**d**) and (**e**).

The SF microparticle scaffolds were also implanted in the rabbit femur as shown schematically in Fig. 8(a). The general physical conditions of the experimental animals including increase in body weight, feeding, behavioral aspects, etc. was found to be normal. Macroscopically, there was no hemorrhage, encapsulation, discoloration, necrosis or infection at the implant site at any observation period. After one week of implantation (data not shown), minimal quantity of new bone formation was noted at the defect edge and a gap/void between the implant surface and bone was noticeable. Also, mild degree of fibrosis was observed at the interface and this was comparable to that observed in a control group of animals implanted with a positive control.

After four weeks of implantation, new woven bone arising from periosteum and endosteum formed along the implant surface and it completely filled the gaps/void at the interface. Refer Fig. 8(b,c). Also, formation of loosely packed woven bone was observed between the SF microparticles in the scaffold and in other places the gaps in the scaffold were filled with non-osseous tissue. Mild degree of inflammation was also observed around the implant. Several other authors have also observed such mild inflammation around SF scaffold *in vivo* implantations and it is known to subside over a period of time^38^.

Thus, in this study, we have demonstrated a biocompatible SF scaffold with superior mechanical properties, interconnected pores and appropriate porosities with slower rate of bioresorption. The scaffold was prepared by fusing together SF microparticles. We have demonstrated a novel method to prepare silk fibroin microparticles using a hexafluoroisopropanol (solvent) - methanol (coagulant) combination. The SF microparticles formed using this process are mono-disperse, spherical, non-porous and highly crystalline. The particle sizes obtained using this process are of the order of few hundreds of microns. We also showed that these particles could be aggregated together in random close packing in a mold. A novel step of gluing these aggregated microparticles using the dilute silk fibroin solution has also been demonstrated. The biocompatibility has been shown using both *in-vitro* and *in-vivo* techniques. We also show that the scaffold supports cell adhesion, proliferation of MG63 osteoblast-like cells, along with mineralization of scaffolds. *In vivo* bone implantation studies have shown formation of woven bone around the implant and in several cavities within the implant after only 4 weeks of implantation. These properties make the scaffold a promising candidate for bone regeneration.

## Materials and Methods

### Preparation of Regenerated Silk Fibroin Solutions

The bivoltine *Bombyx mori* cocoons (CSR 2 breed obtained from Central Sericultural Research and Training Institute, Mysore) were used as supplied. The cocoons were boiled in 0.5 w/v% of NaHCO_3_ (Merck) solution twice for 30 minutes each to remove sericin. The extracted fibroin mass was dried at 60 °C for two days and then dissolved in 9.3 M Lithium Bromide (Sigma Aldrich) solution at 60 °C for 4 h. This silk fibroin - LiBr solution was then extensively dialyzed for 48 hat 4 °C to ensure complete removal of the salt. This Regenerated Silk Fibroin (RSF) solution so obtained was then centrifuged at 12000 rpm for 20 minutes at 4 °C. RSF was lyophilized at −55 °C for 6 hours to obtain SF powder, which was used for further experiments.

### Preparation of SF microparticles

**Figure 9:**
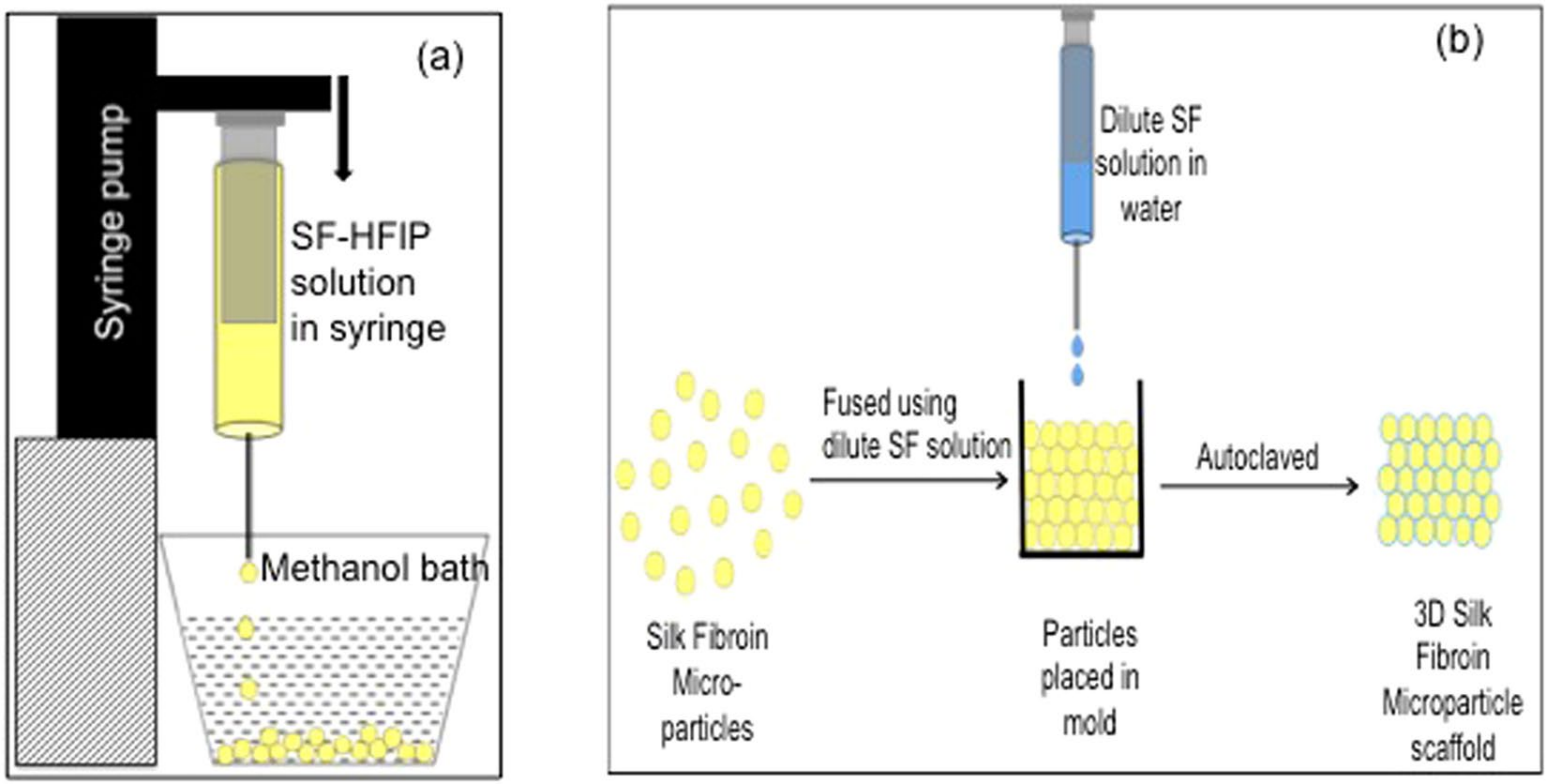
(**a**) Schematic for preparation of silk micro-particles and (**b**) Schematic of Scaffold making process.

Solution of 3wt/v% concentration of lyophilized SF powder in Hexafluroisopropanol (HFIP) (Sigma Aldrich) was prepared. The solution was then loaded in a disposable plastic syringe equipped with a 20 G needle and used for preparation of SF microparticles. The syringe was mounted on an infusion pump from Harvard Apparatus (Model PHD 2000) and a flow rate of 1 ml/min was used. The drops of SF-HFIP solution were allowed to fall into a methanol coagulant bath as shown in Fig. 9(a). The particles so formed were kept immersed in the methanol bath for 15 h. This methanol was then removed and the particles were allowed to air-dry for 1 h. The particles were further vacuum dried at 60 °C for 12 h. The particles were imaged under a light microscope and representative images were captured. Particle size analysis was done using ImageJ software and at least 100 particles were analyzed to report an average particle size value.

### Fourier Transform Infrared (FTIR) Spectroscopy

FTIR study was conducted on SF micro-particles using a Perkin Elmer (Spectrum GX) coupled with Golden Gate Diamond ATR. 32 scans were recorded using a resolution of 4 cm^−1^ in the range of 1400 to 1800 cm^−1^. The amide I peak obtained in the spectral regime of 1580 to 1720 cm^−1^ was used for analyzing the secondary structure of fibroin protein. The spectrum was smoothened using a Savitsky – Golay 9 point method and Peakfit v4.1 software was used for further analysis. 12 peaks were fitted after deconvolution using the second derivative method as per the protocol described by author Hu *et al*. (1). The curve fitting was then done automatically till an r^2^ value greater than 0.99 was obtained. We have defined “Crystallinity Index” as the ratio of absolute areas of crystalline beta sheet peaks (1616–1621, 1622–1627, 1628–1637 cm^−1^) to ratio of random coil peaks and alpha helix peaks (1638–1646, 1647–1655 and 1656–1662 cm^−1^).

### Preparation of Scaffold

Accurately weighed SF micro-particles were poured in a cylindrical mold and 3 wt% of RSF solution was added. The mold was then placed in vacuum oven for 1 hour at 60 °C for drying. The scaffold so formed was then removed from the mold and autoclaved at 121 °C for 20 minutes at 15-psi pressure using a Tuttnauer autoclave (Model 3870 EL) to ensure crystallization of the RSF solution poured onto the particles. See schematic diagram in Fig. 9(b).

### Micro X-ray computed tomography

Micro-computed tomography was conducted on SF Scaffolds using a Scanner type 10 desktop Micro-CT (µCT-40, Scanco Medical, Basseldorf, Switzerland) equipment. The SF scaffolds were scanned at 12 µm voxel resolution with an integration time of 300 ms, at 45 keV with 177 µA current. 2D slices were compiled and analyzed to render 3D images to obtain quantitative architectural parameters. Morphometric indices determined from representative areas were used to calculate bulk porosity. The SF scaffold images were also inverted to analyze pore size distribution by quantifying the number and size of pore space domains. This scan data was further analyzed to provide information on pore interconnectivity.

### Porosity estimation

Theoretical porosity of SF scaffold was calculated using following formula:

1 $$P=(\frac{{V}_{m}-{V}_{s}}{{V}_{m}})\ast 100$$

where *V*_*m*_ = *πr*^2^*h* and *V*_*s*_ = *w/ρ* such that *r* is radius of scaffold in cm and *h* is height of cylindrical scaffold in cm, *w*_*s*_ is weight of scaffold in g and *ρ* is density of SF = 1.35 g/cc.

### Scanning Electron Microscopy (SEM)

SEM analysis was performed on the SF micro-particles and the scaffold using a Quanta 200 3Dmachine equipped with a tungsten filament. The micro-particles and the scaffold were coated with a thin conducting layer of Au prior to imaging. Representative micrographs at appropriate magnifications were captured. Also, for imaging the cut sections, the SF micro-particles were immersed in Liquid Nitrogen for half an hour and then cryo-fractured using a pestle. These cryo-fractured samples were loaded onto the SEM stub and used for imaging.

### Mechanical Testing

The cylindrical scaffolds, 8 mm in diameter and 5 mm in height, prepared using the earlier described protocols were subjected to unconfined uniaxial compression loading using a Bose Electroforce 3200 Series III machine equipped with a 450 N load cell. The compression speed was set at 0.005 mm/sec and experimental parameters used for testing included a preset load of −1 N and scan time of 40 seconds. Wet modulus was also determined by soaking the scaffolds in 5 ml of PBS for 12 hours prior to measurement. The time duration of 12 h was determined from our swellability experiments, (see Supplementary Fig. S5) which show that the scaffold predominantly swells during the first 6 h after immersion in PBS, and the weight of the scaffold later remains constant. The soaked scaffolds were patted dry using lint-free tissue and subjected to compression test as per the protocol described above. A stress-strain graph was plotted. The compression modulus is reported as the peak obtained in the compression modulus vs strain graph as per the protocol described by Li *et al*.^37^.

### Degradation Studies

*In vitro* degradation studies were conducted on the cylindrical silk fibroin scaffold. Protease XIV enzyme (Sigma Aldrich) was used for this study. The scaffolds were incubated (Incubator: New Brunswick Scientific, Innova CO 48) at 37 °C in 2 U/ml of freshly prepared enzyme solution in Phosphate Buffered Saline (Himedia) for 7 days under sterile conditions and the weight loss was monitored after 2, 4 and 7 days degradation. The enzyme solution was replaced after every 24 h to prevent any loss of activity of the enzyme. The scaffolds were dried in vacuum oven at 60 °C for 2 hours before recording the weight.

### *In-vitro* and cytotoxicity studies

*In-vitro* cytotoxicity studies were performed according to ISO 10993-5 guidelines. L-929 fibroblast cell line was procured from National Center for Cell Sciences, Pune. Cells were routinely cultured and maintained in DMEM (Gibco), containing 10% FBS (Gibco, heat inactivated) at 5% CO_2_. Cells were harvested from a near confluent flask by trypsinization, suspended in complete media and seeded at a density of 10,000 cells/well in a sterile 96 well plate tissue culture plate. Cells were allowed to grow at 37 °C, at 5% CO_2_, for 24 hours. MTT assay was carried out for both direct contact and indirect contact methods and absorbance of color formed was measured, as the end point of the assay.

#### Direct contact

In direct contact, a sterilized silk scaffold (weight: 32 mg) was gently placed on the monolayer of L929 cells. This was further allowed to incubate for another 24 hours, keeping other incubation conditions constant. The scaffolds were removed using forceps, and MTT (Sigma Aldrich, India (working solution 0.5 mg/ml)) solution was added. Cells were allowed to incubate with MTT for 4 hours, followed by addition of DMSO (HiMedia) as solubilization reagent. Plates were read at 570 nm, on spectrophotometer and %viability was calculated as the ratio of absorbance in plate without scaffold to absorbance in plate with scaffold.

#### Indirect contact

Scaffolds were incubated overnight, in 200 µl of complete media. The incubation was carried out at 37 °C, for 24 h. Followed by the incubation, the extract was used to replace media in the well where the cells were seeded 24 hours before. Incubation at 37 °C, 5% CO_2_, was continued for 24 hours. The % viability was calculated using MTT assay as described above.

### *In-vitro* studies on osteoblast culture in the SF scaffold environment

The osteosarcoma cell line MG63 was procured from National Centre for Cell Science, Pune. Cells were routinely cultured and maintained in alpha-MEM (Sigma Aldrich) + 10% FBS (Heat inactivated, Gibco) at 37 °C and 5% CO_2_.

#### Osteoblast psysiological markers estimation

MG63 (5 × 10^4^ cells/well) cells were seeded on scaffold and incubated in alpha-MEM culture media containing 10% mesen FBS (Gibco) for 24 hrs, at 37 °C, 5% CO_2_. After 24 h, routine media was replaced by osteogenic medium (Stem-pro osteogenic media, Gibco) and cultured for 21 days. Osteogenic media was replenishment at every 48 hours. Expression of osteoblast activity markers was evaluated by estimation of alkaline phosphatase activity and Ca^2+^ deposition (Alizarin Red S staining) on day 7, 14 and 21.

#### Estimation of alkaline phosphatase (ALP) activity

ALP activity was estimated using colorimetric ALP assay kit (Abcam, UK). MG63 cells were cultured on scaffold as described above. Conditioned media was collected on day 7, 14 and 21. Standard assay for ALP was carried out as per manufacturer’s instruction. For measuring ALP expression in cultured MG63 cells, 80 µL of the spent media was incubated with 50 µL of p-nitrophenyl phosphate (5 mM) solution at room temperature for 1 h in the dark. At the end of the incubation, enzyme activity was stopped by 20 µL of stop solution. Absorbance was measured at 405 nm. Standard graph was then used to estimate ALP activity in samples.

#### Extracellular Ca^2+^ deposition and quantitation

Ca^2+^ deposition was estimated using Alizarin Red S staining. MG63 cells were cultured on scaffold as described above for 21 days. On days 7, 14 and 21 Alizarin Reds S staining was carried out. For staining scaffolds were fixed with 10% formalin for 10 minutes at room temperature. Post fixation scaffolds were washed with 1X PBS twice. Scaffolds were stained with 2% Alizarin Red S solution (Sigma) for 30 minutes at room temperature. After staining scaffolds were washed with distilled water. Extraction of deposited Ca^2+^ was carried out in 0.5 ml 10% Acetic acid for 30 min at room temperature and 85 °C for 10 minutes. Supernatant was collected after centrifugation at 20,000 g for 15 minutes. Absorbance of supernatant was estimated at 405 nm. Scaffolds without cells were used as background control.

### *In-vivo* studies

*In-vivo* experiments were carried out to assess any acute inflammatory response from the scaffold. All procedures of the study were in accordance with the standard operating procedures of the PRADO and the guidelines set by the Committee for the Purpose of Control and Supervision of Experiments on Animals (CPCSEA) as published in The Gazette of India, December 15, 1998. All protocols were approved by the Institutional Animal Ethics Committee of PRADO Pvt. Ltd., where our *in-vivo* studies were carried out. Details of the methods and results are part of the supplementary information.

We also carried out bone implantation studies at Sree Chitra Tirunal Institute for Medical Sciences, Thiruvananthapuram, India after the Institutional Animal ethics committee approval (SCT/IAEC-184/January/2016/89). Animals handling and care were as per the regulations complying with the CPCSEA guidelines. The implantation procedure was carried out in New Zealand white rabbits under clean and aseptic conditions as per the ISO 10993-6:2007 guidelines in accordance with OECD principles of GLP. The rabbits were anaesthetized using Ketamine (80 mg/kg) and Xylaxin (5 mg/kg of body weight). After cleaning with 70% alcohol and application of the betadine solutions, three holes were drilled using low speed drill with profuse irrigation with saline. After implantation of test materials and positive controls (titanium pins), the wound was closed using sutures. The study was conducted in 8 rabbits, 4 animals each for one and four weeks. At the end of the implantation study, animals were euthanized by an overdose of anesthetic agent. The sites of implantation were then macroscopically evaluated for tissue reactions and the implant materials along with femur bone were collected for histo-pathological evaluation after being fixed in 10% formalin.

## Electronic supplementary material

Supplementary information
