# Supplementary material for: Silk fibroin micro-particle scaffolds with superior compression modulus and slow bioresorption for effective bone regeneration
Source: Sci Rep. 2018 May 8;8:7235. doi: 10.1038/s41598-018-25643-x (PMC5940924; doi:10.1038/s41598-018-25643-x)
Supplement: Supplementary file 1 — Supplementary information [file 41598_2018_25643_MOESM1_ESM.doc]

**Electronic Supplementary Information**

Silk fibroin micro-particle scaffolds with superior compression modulus and slow bioresorption for effective bone regeneration

Anuya Nisal^a^,*, Raeesa Sayyad^b^, Prachi Dhavale^a^, Bhakti Khude^a^, Rucha Deshpande^b^, Vidhyashri Mapare^b^, Swati Shukla^b^, Premnath Venugopalan^a^

^a^ – Polymer Science and Engineering Department, National Chemical Laboratory, Pune -411008,

^b^ – BiolMed Innovations Pvt. Ltd., 100, NCL Innovation Park, Dr. HomiBhabha Road, Pune – 411008.

Tel: 91 20 25903013; Email:[aa.nisal@ncl.res.in](mailto:aa.nisal@ncl.res.in)

Supplementary Figure S1. Thermogravimetric analysis on SF microparticles


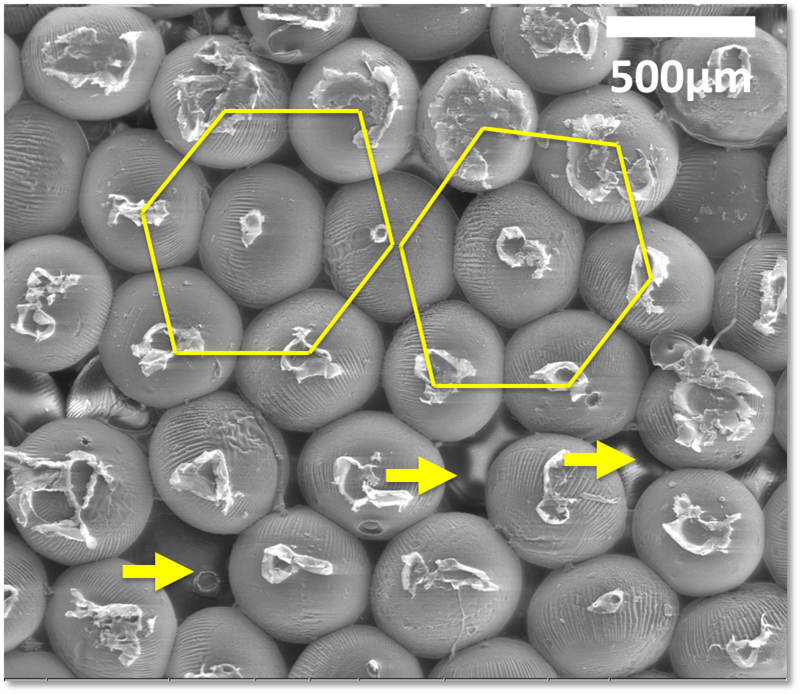


Supplementary Figure S2. SEM micrograph of SF microparticle scaffold (top surface). Arrows point to defects in hexagonal packing

Supplementary Figure S3. Pore size distribution in SF microparticle scaffold obtained from Micro CT experiment

*In-vivo* experiments were carried out to assess any acute inflammatory response from the scaffold. All procedures of the study were in accordance with the standard operating procedures of the PRADO and the guidelines set by the Committee for the Purpose of Control and Supervision of Experiments on Animals (CPCSEA) as published in The Gazette of India, December 15, 1998. All protocols were approved by the Institutional Animal Ethics Committee of PRADO Pvt. Ltd. Scaffolds (2mm X 6mm) were subcutaneously implanted in Sprague Dawley (SD) rats. The study included 4 rats, 2 male, 2 female weighing 200-250g and the age group was restricted to 6-7 weeks. Animals were fed with commercially available standard diet and water. On the day of implantation, animals were anesthetized using standard Isoflouran protocol. Skin at the site of surgery was cleaned and shaved. The test material were implanted subcutaneously in lateral subcutaneous pockets. The skin was then wiped clean with topical disinfectant. Animals were maintained on routine food and water and observed for a week. Incision region was coarse monitored and no deaths were reported during the time of the experiment. At the end of study period, on the 7^th^ day, animals were subjected to euthanasia. After gross anatomical observation, implant were recovered and skin tissue under and around implant was examined for any inflammatory response by standard histopathology procedures. Histological sections were studied for local inflammatory response at the implant tissue interface. The histopathology images were analyzed for hemorrhages, fibrous tissue proliferation and infiltration of multinuclear cells.

The histopathology studies on the tissue surrounding implant were evaluated for fibrous tissue formation, multinuclear cell infiltration and hemorrhages as shown in Figure S4. A mild inflammatory response at the site of implantation was found at the implant tissue interface. The presence of mild inflammation at the implantation site for first few days after implantation has been observed for other SF scaffolds also ^37^. Also, this response has been shown to subside over time and support growth of new tissue. Further *in-vivo* studies are required to ascertain the suitability of these scaffolds for BTE applications.


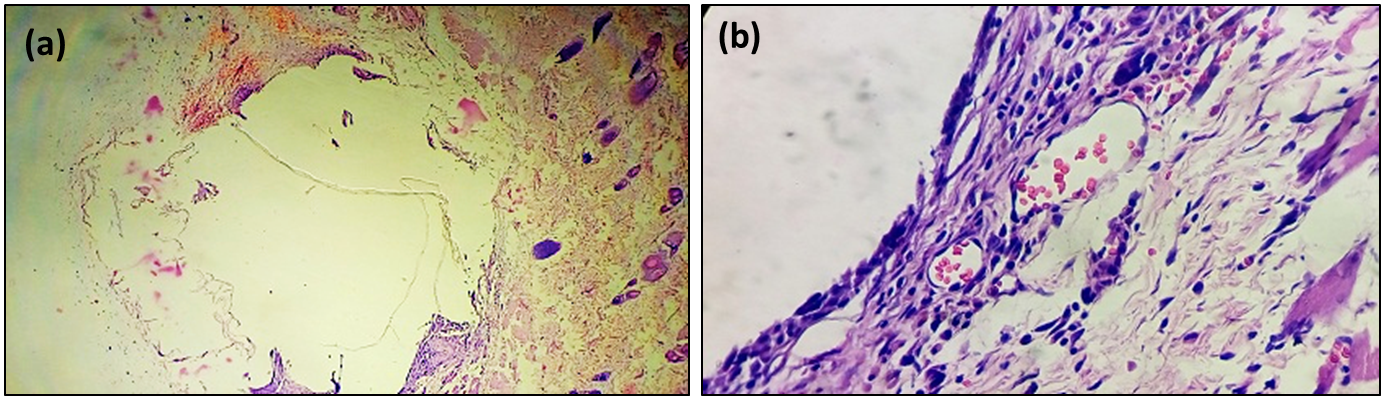


**Supplementary Figure S4.** HE stained tissue surrounding SF microparticle scaffold at low (a) and high (b) magnifications post 7 days of implantation


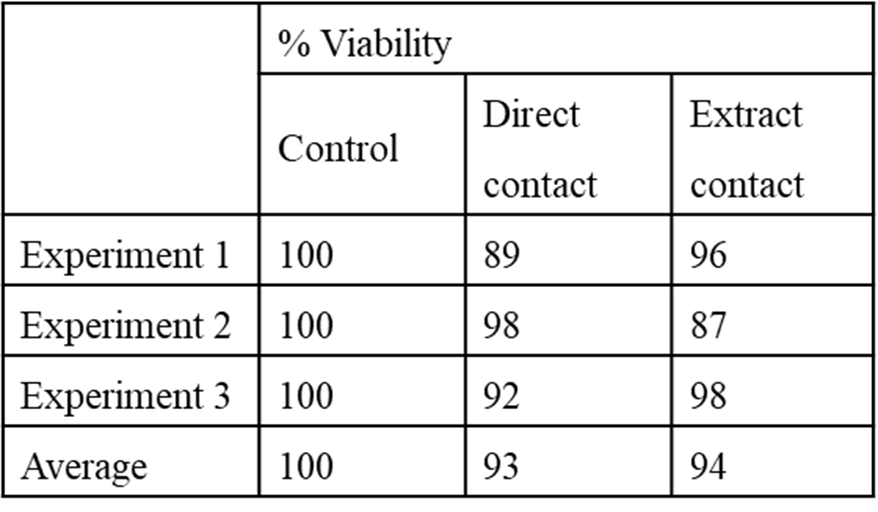


**Table S1.** % Biocompatibility studies as per ISO 10993-5 guidelines for SF microparticle scaffold


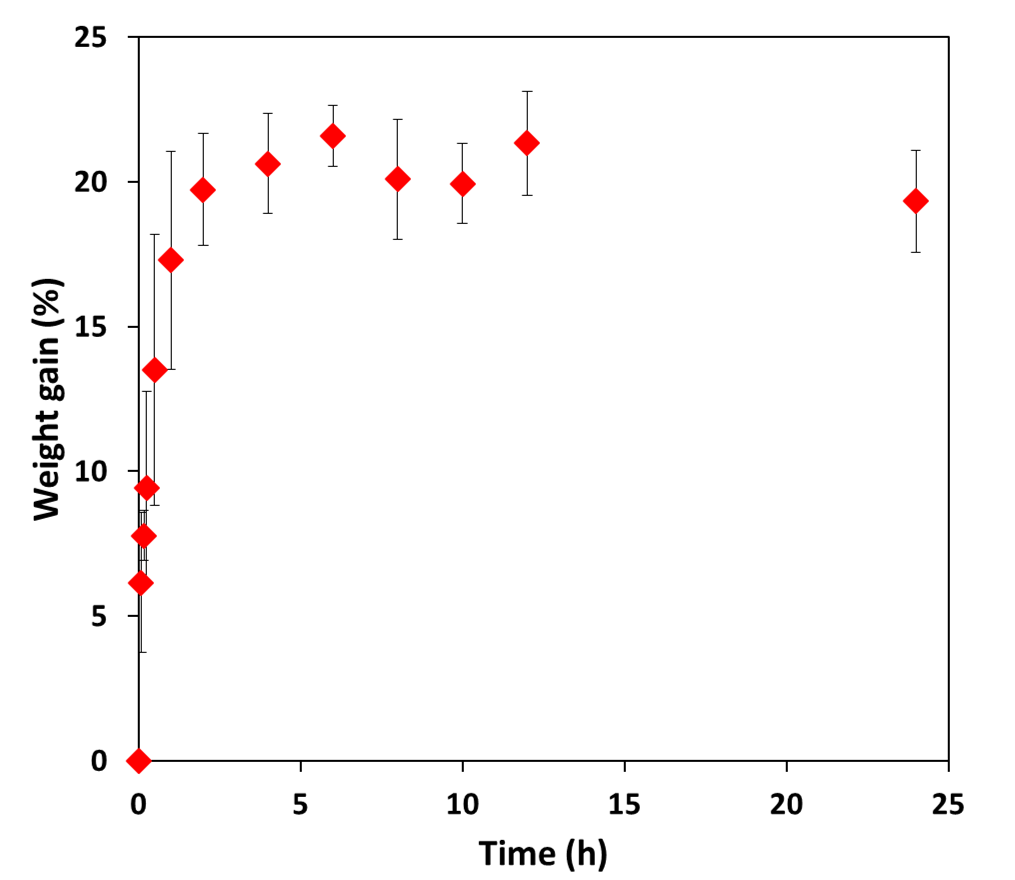


Supplementary Figure S5. % weight gain as a function of time in PBS for SF microparticle scaffold
